# Differential effects of periodontal microbiome on the rheumatoid factor induction during rheumatoid arthritis pathogenesis

**DOI:** 10.1038/s41598-022-21788-y

**Published:** 2022-11-16

**Authors:** Ji-Won Kim, Hyerin Jung, In-Pyo Baek, Yoojun Nam, Jaewoo Kang, Min Kyung Chung, Jun-Beom Park, Jennifer Lee, Seung-Ki Kwok, Wan-Uk Kim, Sung-Hwan Park, Ji Hyeon Ju

**Affiliations:** 1 Division of Rheumatology, Department of Internal Medicine, Daegu Catholic University School of Medicine, Daegu, Republic of Korea; 2 Division of Rheumatology, Department of Internal Medicine, College of Medicine, Seoul St. Mary’s Hospital, The Catholic University of Korea, 222 Banpo-daero, Seocho-gu, Seoul, 06591 Republic of Korea; 3 YiPSCELL Inc., Seoul, Republic of Korea; 4 Division of Rheumatology, Department of Internal Medicine, Ewha Womans University College of Medicine, Seoul, Republic of Korea; 5 Department of Periodontics, College of Medicine, The Catholic University of Korea, Seoul, Republic of Korea

**Keywords:** Rheumatology, Rheumatic diseases, Immunopathogenesis

## Abstract

Association between exposure to periodontal bacteria and development of autoantibodies related to rheumatoid arthritis (RA) has been widely accepted; however, direct causal relationship between periodontal bacteria and rheumatoid factor (RF) is currently not fully understood. We investigated whether periodontal bacteria could affect RF status. Patients with preclinical, new-onset, or chronic RA underwent periodontal examination, and investigation of subgingival microbiome via 16S rRNA sequencing. Degree of arthritis and RF induction was examined in collagen-induced arthritis (CIA) mice that were orally inoculated with different periodontal bacteria species. Subsequently, single-cell RNA sequencing analysis of the mouse spleen cells was performed. Patients with preclinical RA showed an increased abundance of the Porphyromonadacae family in the subgingival microbiome compared to those with new-onset or chronic RA, despite comparable periodontitis severity among them. Notably, a distinct subgingival microbial community was found between patients with high-positive RF and those with negative or low-positive RF (*p*=0.022). Oral infections with the periodontal pathogens *P. gingivalis* and *Treponema denticola* in CIA mice similarly enhanced arthritis score, but resulted in different levels of RF induction. Genes related to B cell receptor signaling, B cell proliferation, activation, and differentiation, and CD4^+^ T cell costimulation and cytokine production were involved in the differential induction of RF in mice exposed to different bacteria. In summary, periodontal microbiome might shape RF status by affecting the humoral immune response during RA pathogenesis.

## Introduction

Rheumatoid arthritis (RA) is a complex autoimmune disease, resulting from interactions between patients’ genetic background and environmental triggers. Periodontitis is considered as an environmental trigger for RA, and *P. gingivalis*, a major periodontal pathogen, can drive autoimmune responses and arthritis development^1^. *P. gingivalis* oral infection induced periodontitis and autoimmune arthritis in experimental animal models^2,3^. Lewis rats exposed to *P. gingivalis*, not those exposed to control gel, developed joint inflammation and destruction^2^. A previous study from our group found that *P. gingivalis* infected collagen-induced arthritis (CIA) mice showed more severe arthritis than noninfected CIA mice, combined with a greater protein citrullination in the joints^3^. Our group also found in a subsequent study that interrupting *P. gingivalis* oral infection by anti-fimbriae antibody recovered the increased autoimmune arthritis by *P. gingivalis* infection^4^. These results indicate that periodontitis and *P. gingivalis* have important roles in the pathogenesis of RA.

Periodontitis is prevalent in patients with established RA; however, it is already present in patients with early or preclinical RA, showing more frequent and more advanced pathogenesis than controls^5–8^. Scher and colleagues found that the subgingival microbiota of patients with early RA are distinct from those of controls, although the microbiota deviations were dependent on the presence and severity of periodontitis^9^. In contrast, distinct subgingival microbiota were found in periodontally healthy patients with RA when compared with those in controls, thus indicating that the changes are RA-specific^10^. A recent study demonstrated alteration of the subgingival microbiota with an increased abundance of *P. gingivalis* in both periodontally healthy and diseased sites of anti-citrullinated protein antibodies (ACPA)-positive individuals at risk of RA^11^. Taken together, these suggest that the periodontal microbiota changes precede the onset of RA, irrespective of periodontitis severity.

Autoantibodies related to RA, such as rheumatoid factor (RF) and ACPA, appear in the body prior to the onset of RA^12–14^. Titers for RF and ACPA have been reported to markedly increase during the preclinical phase^13,14^. Furthermore, serum antibodies against *P. gingivalis* increased during the preclinical phase of arthritis and became stable after RA diagnosis^15^. Therefore, an association between periodontal bacteria exposure and RA autoantibody development can be suggested. However, compared to well-studied mechanisms such as in the case of the association between periodontal pathogens and ACPA^1,10,16,17^, the role of periodontal pathogens in RF status is not fully understood in RA. Some studies have shown a positive relationship between periodontal bacteria and RF, whereas others have not^18,19^. These conflicting results might originate from limitations of these studies–either from only investigating small samples of bacteria or from not using high-throughput sequencing techniques to measure bacterial load.

In this study, we investigated the subgingival microbiome in patients of preclinical RA (pre-RA), new-onset RA (NORA), and chronic RA groups based on metagenomic sequencing (along with their periodontitis status). We then compared the subgingival microbiome according to each RF status to explore the association between periodontal bacteria and RF. Furthermore, murine model experiments and single-cell RNA sequencing analyses were performed to confirm the potential causal link between periodontal bacterial infection and RF induction.

## Methods

### Patients

The study protocol was approved by the Institutional Review Board of Seoul St. Mary’s Hospital, Catholic University of Korea (Approval number: KC15RISI0612) and all research was performed in accordance with relevant guidelines and regulations. Study subjects were enrolled from the Rheumatology Center of Seoul St. Mary’s Hospital. Patients with pre-RA, NORA, and chronic RA were consecutively enrolled after written informed consent was obtained from each of the subjects. Pre-RA was defined if subjects had positive RF and/or ACPA, but physical examination and musculoskeletal ultrasound did not show evidence of definite synovitis. Patients with NORA and chronic RA were those who met the American College of Rheumatology and European League Against Rheumatism classification criteria for RA at the time of diagnosis^20^: patients with NORA had a disease duration of less than 6 months, whereas patients with chronic RA had the condition for over 6 months. The exclusion criteria included antibiotic use within 3 months, as well as any medical conditions that required antibiotic use. Microbiome analysis was performed in the patients who agreed to the subgingival plaque sampling. The characteristics of the study population who underwent periodontal examination and subgingival microbiome analysis are described in Supplementary Table S1 and S2, respectively.

### Periodontal examination and sample collection

Periodontal probing depth (PPD) was measured from the participants in this study by inserting the periodontal probe into the space between the tooth surface and gingiva. The distance from the free gingival margin to the base of the gingival sulcus was defined as the PPD. Subgingival plaque samples were collected from the deepest interproximal sites using a periodontal curette. Samples were placed in a microtube and frozen and stored at –80 ℃ until further analysis.

### Next-generation sequencing and bioinformatics analysis pipeline

Bacterial genomic DNA was extracted from plaque samples. Sequencing targeted the V3-V4 region of the 16S rRNA gene from the extracted DNA. The 16S gene was amplified using the forward primer 341F (5′-TCGTCGGCAGCGTC-AGATGTGTATAAGAGACAG-CCTACGGGNGGCWGCAG-3′; underlined sequence indicates the target region primer) and reverse primer 805R (5′-GTCTCGTGGGCTCGG-AGATGTGTATAAGAGACAG-GACTACHVGGGTATCTAATCC-3′). Then, secondary amplification for attaching the Illumina NexTera barcode was performed with an i5 forward primer (5’-AATGATACGGCGACCACCGAGATCTACAC-XXXXXXXX-TCGTCGGCAGCGTC-3’; X indicates the barcode region) and i7 reverse primer (5’-CAAGCAGAAGACGGCATACGAGAT-XXXXXXXX-GTCTCGTGGGCTCGG-3’). The polymerase chain reaction (PCR) product was confirmed and visualized using a Gel Doc System (Bio-Rad, Hercules, CA, USA) on a 1% agarose gel electrophoresis. The amplified products were purified using CleanPCR (CleanNA, Waddinxveen, Netherlands). Equal concentrations of purified products were pooled together, and short fragments (non-target products) were removed with CleanPCR (CleanNA). Quality and product size were assessed on a Bioanalyzer 2100 (Agilent, Palo Alto, CA, USA) using a DNA 7500 chip. Mixed amplicons were pooled, and the sequencing was carried out at ChunLab, Inc. (Seoul, Korea), using the paired-end method (250 bp x2) with an Illumina MiSeq Sequencing system (Illumina, San Diego, CA, USA) according to the manufacturer’s instructions.

Processing raw 16S rRNA gene sequences starts with filtering out the low-quality sequences using the Trimmomatic 0.32. After quality filtering, two sequences representing each end of the same PCR amplicon were merged using PANDAseq. Primers were trimmed using ChunLab’s in-house program. Sequences were denoised using DUDE-Seq, and identical sequences were de-replicated in this step to reduce the computational time. Denoised and dereplicated sequences were then subjected to taxonomic assignment. We used the USEARCH program (8.1.1861_i86linux32) to search and calculate sequence similarities of the query next-generation sequencing (NGS) reads against the EzBioCloud 16S database. A 16S similarity of 97% was used as the cut-off for species-level identification. The remaining reads were checked for chimera using the UCHIME program and the non-chimeric 16S rRNA database from EzBioCloud. Sequence data were then clustered using CD-HIT and UCLUST.

### Mice

Male, 5-week-old DBA/1J mice were purchased from OrientBio (Seongnam, Korea). The mice were maintained under pathogen-free conditions at the Catholic University of Korea. All experimental procedures were performed in accordance with the Laboratory Animals Welfare Act, the Guide for the Care and Use of Laboratory Animals, and the Guidelines and Policies for Rodent Experiments provided by the Institutional Animal Care and Use Committee of the Catholic University of Korea (Approval number: 2020-0011-01).

### Induction and assessment of collagen-induced arthritis

To induce autoimmune arthritis, DBA/1J mice were immunized with type II collagen (Chondrex, Redmond, WA, USA) emulsified in complete Freund’s adjuvant (Chondrex). Twenty-one days after the first immunization, a booster of type II collagen (Chondrex), emulsified in incomplete Freund’s adjuvant (Chondrex), was injected intradermally into the mice. Arthritis development was assessed two to three times per week. Arthritis score of each paw was graded between 0 and 4 according to the degree of inflammation: 0 = no evidence of erythema and swelling; 1 = mild swelling confined to the tarsals or ankle joint; 2 = mild swelling extending from the ankle to the tarsals; 3 = moderate swelling extending from the ankle to metatarsal joints; 4 = severe swelling encompass the ankle, foot, and digits, or ankylosis of the limb^21^. Total arthritis score was the sum of each arthritis score of all four paws.

### Bacteria strain

*Porphyromonas gingivalis* strain American Type Culture Collection (ATCC) 33277 and *T. denticola* strain ATCC 35405 were purchased from ATCC (Manassas, VA, USA). The bacteria were cultured in a 37 ℃ anaerobic environment (BD GasPak ^TM^ 150 System; BD, Franklin Lakes, NJ, USA).

### Oral infection with periodontal bacteria

Mice were inoculated with 1 × 10^9^ colony-forming units of *P. gingivalis* or *T. denticola* suspended in 30 μL of phosphate-buffered saline (PBS) with 2% carboxymethyl cellulose (Sigma-Aldrich, St Louis, MO, USA) or, for the control, 30 μL of PBS with 2% carboxymethyl cellulose (Sigma-Aldrich) alone^3^. Then, bacteria-containing gels or control gels were directly inoculated into the oral cavity of mice 3 times per week for 3 weeks, starting at 14 days before the first immunization. This was done during establishment of CIA model. Our group previously confirmed the induction of periodontitis after oral inoculation with periodontal bacteria in the same setting^4^.

### ELISA

The concentrations of rheumatoid factor (RF) and anti-citrullinated protein antibodies (ACPAs) were measured in the sera of mice using a mouse RF-IgM ELISA kit (MyBioSource, San Diego, CA, USA) and mouse ACPA ELISA kit (MyBioSource), respectively.

### Mouse splenocytes isolation

Mice were sacrificed to obtain splenocytes. Mouse spleens were harvested and splenocytes were isolated by straining mashed spleen through a 40 µm cell strainer into ice-cold RPMI medium. Red blood cells were lysed by addition of Gibco™ ACK lysis buffer for 10 minutes. The remaining cells were centrifuged at 1,500 rpm for 5 minutes at 4 °C and the supernatant was removed. The splenocytes were washed once more and resuspended at a concentration of 1 × 10^7^ cells/mL to perform single-cell analysis.

### Single-cell RNA sequencing analysis

Following the manufacturer’s recommendations, single-cell capture and cDNA synthesis were performed using the DB Rhapsody Single-Cell Analysis System. Libraries were run multiple times on an Illumina Next-Seq for sequencing. We used R to perform unsupervised clustering of cells with the data of the SC-sequencing Seurat package version 3.2.3^22^. Genes with expression in less than three cells were filtered out. We processed cells with > 200 or < 2500 expressed genes, < 25% of mitochondrial genes, and < 20,000 of UMIs per cell^23^. Then, the variation coefficient of genes was calculated through Seurat arithmetic. We identified 2000 highly variable genes from each sample by using the ‘vst’ method in Seurat, thus enabling us to align the cells originating from different samples. For dimensionality reduction of all data, principal coordinates analysis (PCoA) was performed based on the first 2000 highest alterable genes. A k-nearest neighbor graph was constructed using Euclidean distances in the space of the first 10 significant principal components. The cells were clustered in a picture using the Louvain modularity optimization algorithm. Then, the final outcomes of unsupervised clustering were visualized via t-distributed stochastic neighbor embedding (tSNE) analysis.

### Cell type annotation through marker gene identification in each cluster

We selected upregulated and downregulated genes in each cluster. These were in relation to the other clusters on the basis of the Wilcoxon rank sum test in Seurat’s implementation, with a >0.25 log fold change compared with the other clusters and a *p* <0.05 (eTable 1 in the appendix). The highly differentially expressed genes (DEGs) in each cluster were calculated so they could be manually annotated with their respective cellular identities. The identities were confirmed using scCATCH for automated reference-based annotation^24^.

### Gene ontology enrichment analysis

K-means clustering was performed on variance-stabilized read counts in order to build a heatmap (selected gene set with variable genes) in R. K-mean option and Euclidean clustering distance of both the row and column were applied together alongside the pheatmap function. We annotated the assigned genes of the resulting clusters using gene ontology analysis using the PANTHER (Protein Analysis Through Evolutionary Relationships) classification system (http://geneontology.org)^25^.

### Statistical analysis

PPD and taxonomic relative abundance were compared among different patient groups using the Wilcoxon rank-sum test or Kruskal-Wallis test. Principal coordinates analysis (PCoA) of UniFrac distances and the PERMANOVA test were carried out to compare the microbial community in each group (beta diversity). Differentially present taxa were selected using the linear discriminant analysis effect size (LEfSe). Arthritis scores and RF and ACPA levels in the CIA mouse groups were compared using unpaired t-test. Statistical analyses were performed using GraphPad Prism version 5 (GraphPad Software, San Diego, CA, USA) and EzBioCloud web-based software (https://www.ezbiocloud.net).

## Results

### Periodontitis in patients with pre-RA, NORA, and chronic RA

**Figure 1:**
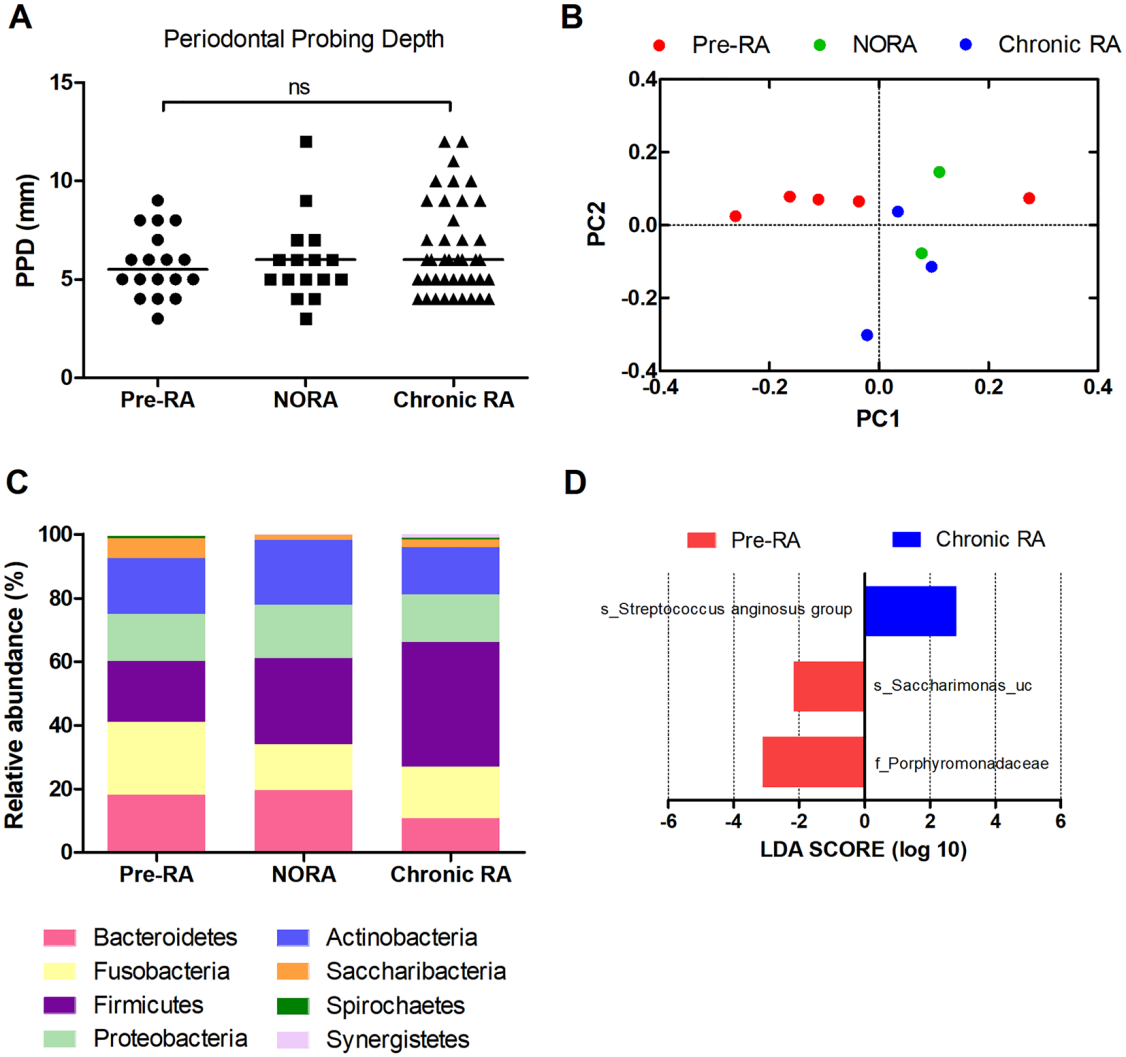
Periodontitis and the subgingival microbiome in patients with preclinical rheumatoid arthritis (pre-RA), new-onset RA (NORA), and chronic RA. (**A**) Comparison of periodontal probing depth (PPD) among patients with pre-RA, NORA, and chronic RA (ns: not significant, Kruskal-Wallis test). (**B**) Beta diversity of the subgingival microbiome between different RA statuses is represented by principal coordinates analysis (PCoA) plot using UniFrac distance matrix (*p* = 0.388, PERMANOVA). (**C**) Comparison of subgingival microbial composition at the phylum level among patients with pre-RA, NORA, and chronic RA (Kruskal-Wallis test). (**D**) Linear discriminant analysis effect size (LEfSe) analysis identifies differentially abundant taxa among patients with pre-RA, NORA, and chronic RA. Red bars and a blue bar indicate taxa abundant in pre-RA and chronic RA groups, respectively.

PPD measurements were conducted in 18 patients with pre-RA, 18 patients with NORA, and 49 patients with chronic RA. PPD did not differ among patients with pre-RA, NORA, and chronic RA (median 5.5, 6, and 6 mm, respectively; *p* = 0.525, Kruskal-Wallis test, Fig. 1A), with all of them showing a similar degree of periodontitis. Next, we examined the prevalence of periodontitis in patients with pre-RA compared to that in controls. Control subjects who did not have RA were matched for age, sex, and body mass index; they were also matched depending on the presence of diabetes or hypertension and smoking status. These data were extracted from the Korea National Health and Nutrition Examination Survey database. When compared to the 72 matched control subjects, patients with pre-RA were more likely to have periodontitis: 44.4% and 50% of patients with pre-RA had a PPD of 4–5 mm and a PPD of ≥6 mm, respectively, whereas 19.4% and 4.2% of control subjects had a PPD of 4–5 mm and a PPD of ≥6 mm, respectively (*p* <0.001, Fisher’s exact test, Supplementary Fig. S1). To summarize, patients with pre-RA more frequently had periodontitis than the controls; moreover, the degree of periodontitis in patients with pre-RA was similar to that of patients with established RA.

### Subgingival microbial composition of patients with pre-RA, NORA, and chronic RA

The subgingival microbiome was investigated in five patients with pre-RA, two patients with NORA, and three patients with chronic RA. As seen in Supplementary Table S2, they were all non-smokers and had high ACPA positivity; however, they had variable degrees of RF titers. Subgingival microbial communities did not significantly differ between RA groups upon PCoA of UniFrac distances (*p* = 0.388, PERMANOVA, Fig. 1B). The relative abundance of phylum level taxa was not significantly different between the RA groups (all *p* >0.1, Kruskal-Wallis test, Fig. 1C). When including all taxonomic levels, differentially abundant taxa among patients with pre-RA, NORA, and chronic RA were identified using LEfSe analysis (Fig. 1D). The Porphyromonadaceae family of bacteria as well as the *Saccharimonas* species were enriched in patients with pre-RA, whereas *Streptococcus anginosus* was only enriched in patients with chronic RA.

### Distinct subgingival microbial composition according to RF status

**Figure 2:**
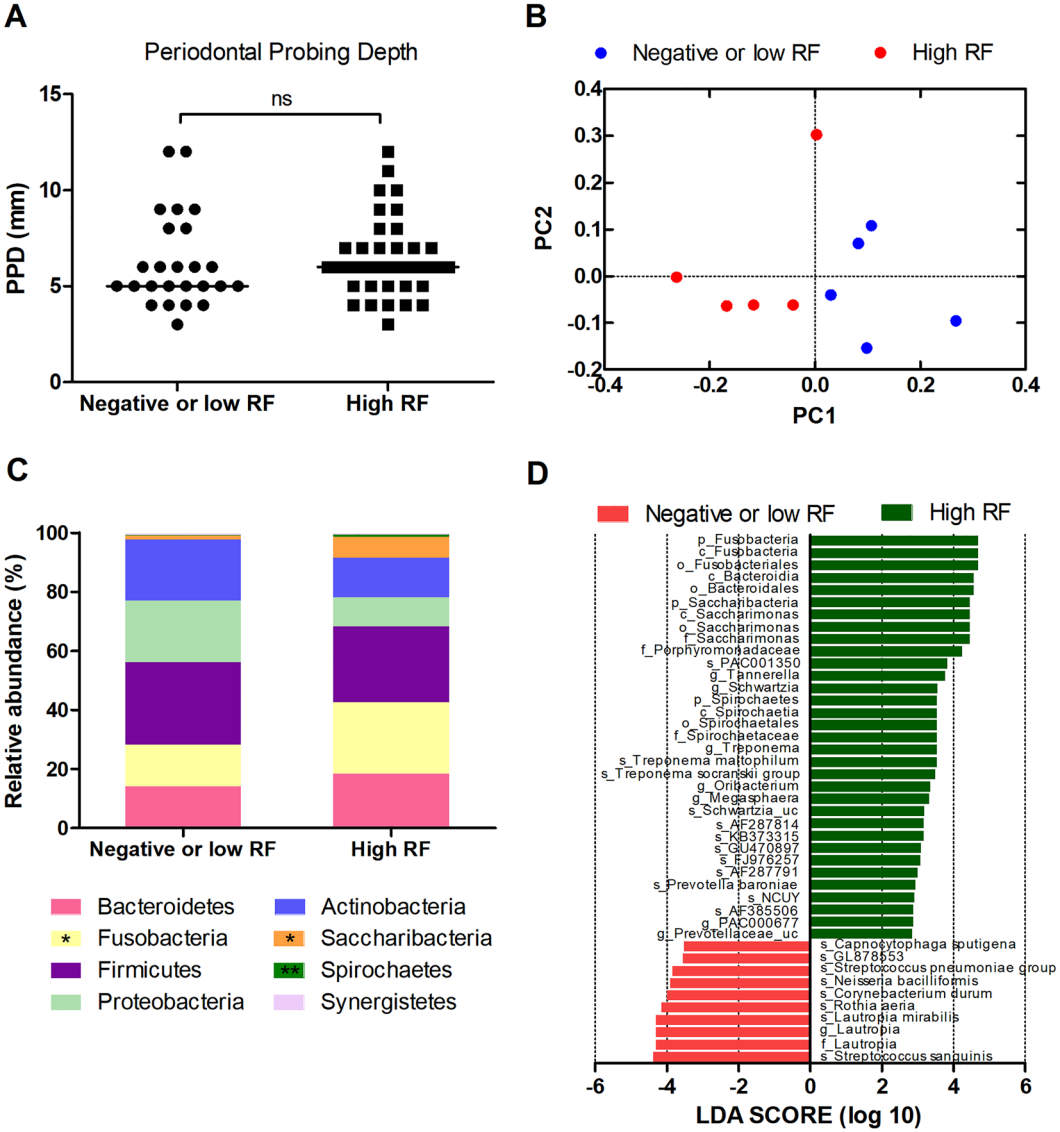
Periodontitis and subgingival microbiome according to rheumatoid factor (RF) status. (**A**) Comparison of PPD between patients with negative or low RF and patients with high RF (ns: not significant, Wilcoxon rank-sum test). (**B**) Beta diversity of the subgingival microbiome between different RF statuses is represented by PCoA plot using UniFrac distance matrix (*p* = 0.022, PERMANOVA). The data were from the same 10 patients who underwent subgingival microbiome analysis. (**C**) Comparison of subgingival microbial composition at the phylum level between patients with negative or low RF and patients with high RF (*p <0.05, **p <0.01, Wilcoxon rank-sum test). (**D**) LEfSe analysis identifies differentially abundant taxa between patients with negative or low RF (red bars) and patients with high RF (green bars).

Patients with pre-RA, NORA, and chronic RA were divided into two groups based on their RF titers: a negative or low RF group and a high RF group. Despite the severity of periodontitis and the PPD not differing between patients with negative or low RF and those with high RF (Fig. 2A), subgingival microbial composition was significantly different between these groups. PCoA of UniFrac distances revealed the clustering of microbial communities according to RF status (*p* = 0.022, PERMANOVA, Fig. 2B). Comparison of taxonomic relative abundance at the phylum level showed that Fusobacteria, Saccharibacteria (formerly TM7), and Spirochaetes were significantly abundant in patients with high RF compared with those in patients with negative or low RF (*p* = 0.047, *p* = 0.028, and *p* = 0.009, respectively, Wilcoxon rank-sum test, Fig. 2C). LEfSe analyses at all taxonomic levels showed 43 differentially abundant taxa between patients with negative or low RF and patients with high RF (Fig. 2D). Specifically, phyla Fusobacteria, Saccharibacteria, and Spirochaetes, families Saccharimonas, Porphyromonadaceae, and Spirochaetaceae were enriched in the high RF group.

### Variable degree of RF induction after inoculating different periodontal bacteria into CIA mice

**Figure 3:**
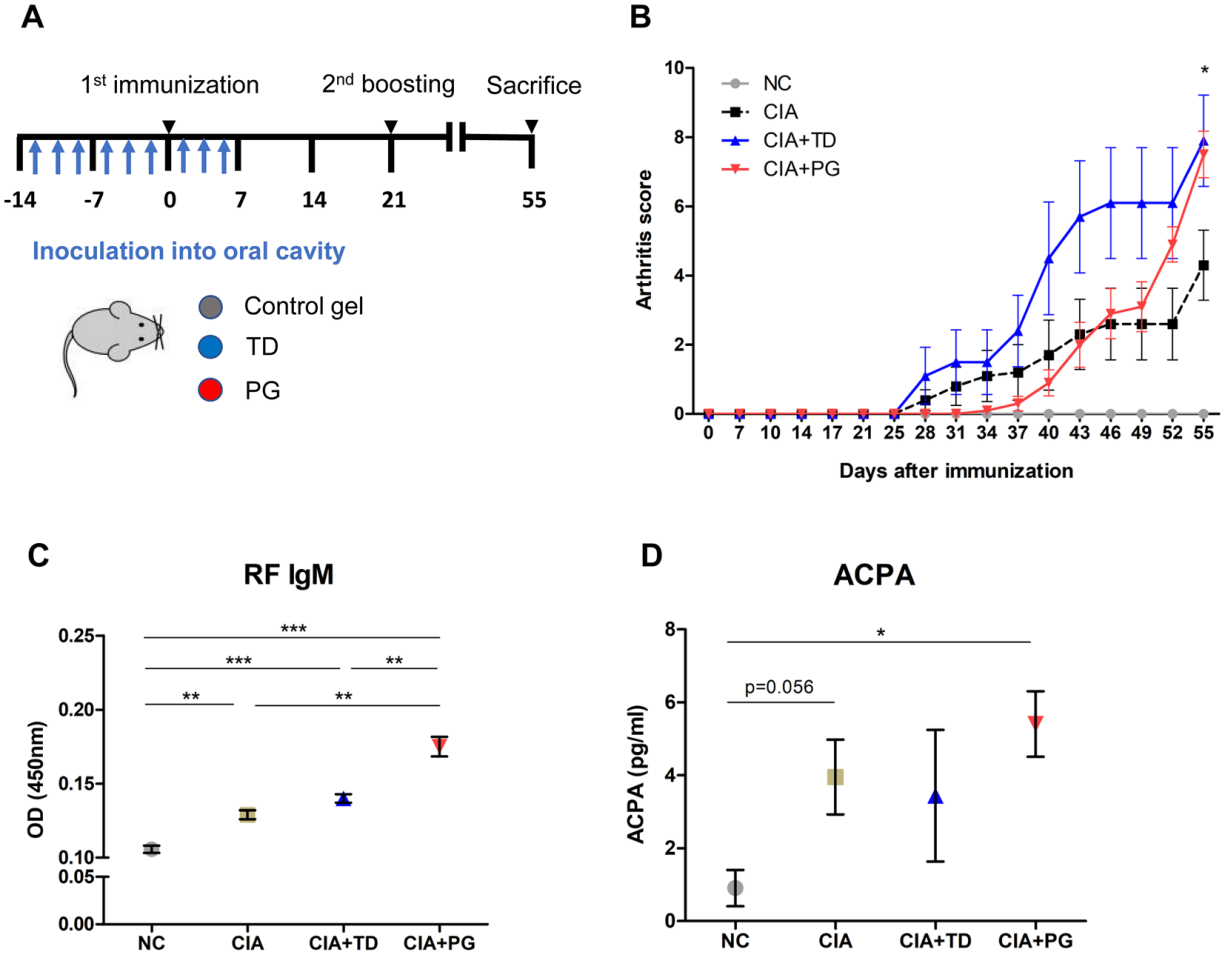
Different degrees of RF induction in collagen-induced arthritis (CIA) mouse models inoculated with different species of periodontal bacteria. (**A**) Schematic illustration of CIA model development and oral inoculation with or without periodontal bacteria, *Porphyromonas gingivalis* and *T. denticola*. (**B**) Changes in arthritis score of normal control mice (n = 5), CIA mice inoculated with control gels (n = 10), CIA mice inoculated with *P. gingivalis*-containing gels (n = 10), and CIA mice inoculated with *T. denticola*-containing gels (n = 10) since first immunization with type II collagen. (**C**) Levels of RF IgM in different mouse groups 55 days after the first immunization (n = 3 in each group). (**D**) Levels of anti-citrullinated protein antibodies (ACPAs) in different mouse groups 55 days after the first immunization (n = 3 in each group). Data are representative of two independent experiments. *p <0.05, **p <0.01, ***p <0.001 by unpaired t test. NC, normal control; PG, *P. gingivalis*; TD, *T. denticola*.

To examine whether different periodontal bacteria could induce different levels of RF, DBA/1J mice were orally inoculated with different bacteria during the establishment of the CIA model. The schematic schedule of oral bacterial inoculation is illustrated in Fig. 3A. Arthritis developed in CIA mouse groups inoculated with control gels and groups inoculated with either *P. gingivalis*- or *T. denticola*-containing gels (Fig. 3B). Fifty-five days after the first immunization, the arthritis score was higher in *P. gingivalis*- and *T. denticola*-inoculated CIA mice than in the control mice, demonstrating that a more severe arthritis developed in the CIA mice inoculated with periodontal bacteria. Although the severity of arthritis did not seem to be significantly different between *P. gingivalis*- and *T. denticola-*inoculated mice, titers of RF were significantly higher in mice inoculated with *P. gingivalis* than in those inoculated with *T. denticola* (*p* = 0.008, unpaired t-test). Titers of ACPA did not differ significantly between *P. gingivalis*- and *T. denticola-*inoculated mice. This indicates different degrees of RF induction according to the species of periodontal bacteria (Fig. 3C,D).

### Transcriptomic differences in spleen-derived B and T cells of *P. gingivalis*- and *T. denticola*-exposed mice

**Figure 4:**
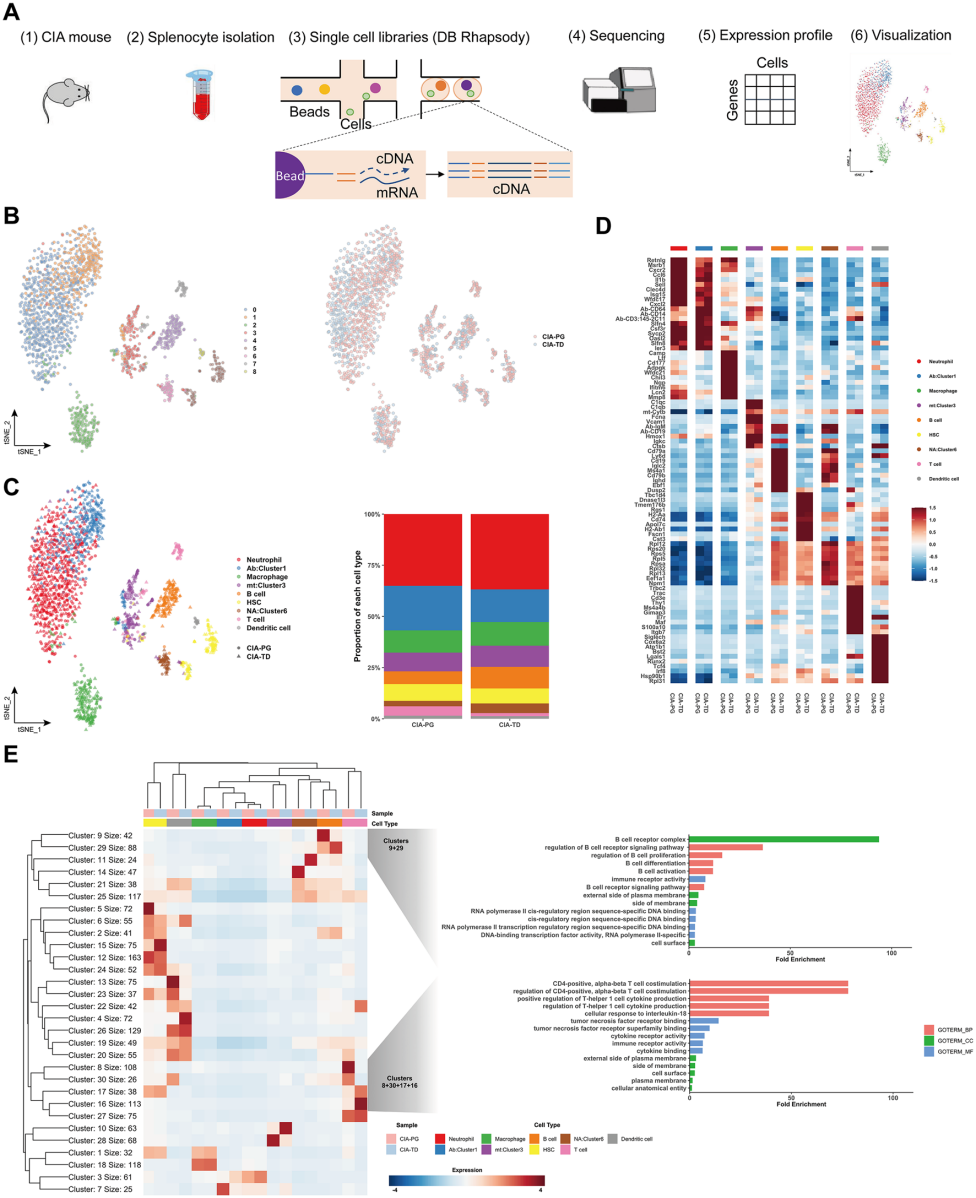
Identification of cell populations and differential gene expression patterns in CIA mice inoculated with different species of periodontal bacteria. (**A**) Schematic illustration of single-cell transcriptome experiments, from splenocyte collection to visualization. (**B**) t-Distributed stochastic neighbour embedding (tSNE) plots of single cells colored by the major cell lineages (left panel) and by the mouse type, CIA-PG or CIA-TD (right panel). (**C**) Identification and proportion of each cell type in the CIA-PG and CIA-TD models visualized by tSNE and bar plots. (**D**) Heatmap of gene expression levels of the 10 most significant genes per cell type clusters. (**E**) Heatmap of K-means clustering of differentially expressed genes (DEGs) and subsequent gene ontology (GO) enrichment analysis. DEGs were clustered into 30 groups (K = 30, the model-based optimal number; left panel). The most differentially enriched GO categories according to the biological process, cellular component, and molecular function are shown for selected clusters in B and T cell populations (right panel). CIA-PG, *P. gingivalis* exposed CIA mice; CIA-TD, *T. denticola* exposed CIA mice; BP, biological process; CC, cellular component; MF, molecular function.

We performed single-cell RNA sequencing of splenocytes of *P. gingivalis*- and *T. denticola*-exposed mice to understand the transcriptomic differences at the single-cell level (Fig. 4A). Three spleens from *P. gingivalis-* and *T. denticola*-exposed mice were obtained for the analysis, respectively. In total, 962 and 851 splenocytes were analyzed after filtration, respectively. First, we mapped a single-cell atlas and defined the cell population of splenocytes. Single-cell atlases of *P. gingivalis*- and *T. denticola*-exposed mice were presented, and the cells were clustered into nine compartments (Fig. 4B). Cell populations such as neutrophils, macrophages, B cells, T cells, and dendritic cells were identified in both groups (Fig. 4C). The pattern and frequency of cell populations were comparable between *P. gingivalis*- and *T. denticola*-exposed mice (*p* = 0.783; Chi-square test). Next, the gene expression of splenocytes was analyzed at a single-cell level. The 10 most significant genes for each cell type were determined, and their expression levels were examined in all cell types (Fig. 4D). The representative genes for each cell type were differentially upregulated in a specific cell type, but not in others, indicating their role in distinguishing each cell type from another. This pattern was observed in both *P. gingivalis*- and *T. denticola*-exposed mice. We then determined the expression levels of differentially expressed genes (DEGs) via K-means clustering (Fig. 4E). Notably, differential gene clusters were found in the B and T cells of *P. gingivalis*- and *T. denticola*-exposed mice. Differential gene clusters of B cells were governed by genes that were related to the B cell receptor signaling pathway and B cell proliferation, activation, and differentiation (Clusters 9 and 29). Differential gene clusters of T cells were governed by genes associated with CD4^+^ T cell costimulation and Th1 cytokine production (Clusters 8, 16, 17, and 30). Detailed results of the gene ontology (GO) enrichment analysis are provided in the appendix. The differential gene expression in B and T cells of mice inoculated with different species of periodontal bacteria might explain their different degrees of RF induction.

## Discussion

The subgingival microbiome of patients with pre-RA showed an increased abundance of the Porphyromonadaceae family when compared to that of patients with NORA or chronic RA, despite similar prevalence and severity of periodontitis. Notably, the subgingival microbial community structure was dependent on the RF status rather than the RA status. Oral infection with different periodontal pathogens, *P. gingivalis* or *T. denticola*, in a murine model of RA resulted in a similarly enhanced arthritis induction but with different degrees of RF induction. In a single-cell RNA-seq analysis of mouse spleen cells, *P. gingivalis*- and *T. denticola*-exposed mice showed highly variable expression patterns of specific genes in B and CD4^+^ T cells. This indicates that periodontal bacteria differentially affect the humoral immune response to induce RF during the evolution of RA.

We demonstrated that periodontitis and changes in the periodontal microbiome already existed in ACPA-positive individuals at risk of RA; the degree and pattern of which were similar to those in patients with new-onset or chronic RA. The unique microbiome change in ACPA-positive individuals at risk of RA was in the increased abundance of the Porphyromonadaceae family, which was in line with previous studies that showed an increase in *P. gingivalis* in individuals at risk of RA^11,26^. In a cohort study consisting of individuals at higher risk of developing RA, serum concentrations of antibody against *P. gingivalis* were significantly higher in the RA-related autoantibody positive group than in the autoantibody negative group, although the presence of periodontitis was similar between these groups^26^. However, concentrations of antibody to other periodontal bacteria did not differ between autoantibody positive and negative groups, indicating that immunity to *P. gingivalis* was associated with RA-related autoantibody production in individuals at risk of RA. A recent study confirmed, via metagenomic sequencing, the dysbiosis and enriched abundance of *P. gingivalis* in the subgingival microbiome of ACPA-positive individuals at risk of RA^11^. Taken together, our results alongside the literature indicate that the oral microbiome changes precede the onset of RA and that *P. gingivalis* plays an important role in this preclinical phase.

We then investigated the role of periodontal pathogens in the development of RA-related autoantibodies, particularly focusing on the RF autoantibody. RF is a diagnostic marker of RA; however, it is also found in other autoimmune and infectious diseases. A previous study identified that RF was present in both the subgingival plaque and serum samples of patients with periodontitis that did not have clinical arthritis, thus suggesting the association between bacterial infection and RF induction^27^. Bacteria colonizing mucosal sites may be a key factor in the induction of B cell activation and autoantibody production. In addition, the levels of autoantibody induction differed depending on the type of bacteria used. For example, oral exposure with *P. gingivalis* induced an ACPA-associated arthritis in rats, whereas oral exposure with *Prevotella intermedia* did not^2^. Our study added the finding that *P. gingivalis*-exposed CIA mice highly induced the RF compared to *T. denticola*-exposed mice. We described the separate and distinct oral microbiota patterns of the high and low RF groups of patients and also confirmed the causal effect of periodontal bacteria on RF induction in a murine model of RA. Therefore, we suggest that periodontal bacteria can differentially influence RF status during RA autoimmunity.

RF is an autoantibody that targets antigenic determinants (epitopes) located on the Fc region of immunoglobulin G (IgG). RF is produced by RF-positive B lymphocytes (expressing RF as a B cell receptor)^28^. Under normal conditions, RF B cells maintain tolerance with autoantigens without producing RF. However, RF B cells can be activated under autoimmune conditions or during microbial infections. Microbial components, such as bacterial lipopolysaccharides (LPS) or DNA, can mediate B cell activation and RF production by a non-specific mechanism. For instance, bacterial LPS or DNA can directly stimulate B cell activation via Toll-like receptor (TLR) signaling^29^. In an earlier study, LPS was injected into several strains of mice to examine their ability to induce RF activity^30^. RF was induced in all strains, except in LPS-resistant mice, and increased RF was paralleled by an increase in other IgM antibodies. Therefore, LPS was considered as a stimulant of polyclonal antibody formation by B cells, thereby inducing RF production. Another recent study has found the positive correlation between LPS-binding protein and RF, suggesting an important role of systemic microbial exposure in development of RF^31^.

However, two additional antigen-specific mechanisms are required to explain the enhanced B cell activation and RF induction after microbial exposure, besides the non-specific B cell activation^32^. First, the synergy between B cell receptors and TLRs. Second, the T cell help. Interaction of B cell receptors and TLRs on B cell surface was enabled by the formation and cross-linking of microbial antigen-IgG immune complexes. The immune complexes signal both B cell receptors and TLRs to activate the RF-producing B cells. B cell receptor signaling can trap immune complexes and concentrate microbial antigens on B cell surface, thus enhances TLR signaling. Selective inhibition of B cell receptor signaling without interfering TLR signaling resulted in a blockage of RF B cell proliferation induced by immune complexes. Next, microbial antigen specific CD4^+^ T cell help is required to increase RF production. After microbial antigens are internalized and processed into antigenic peptides in RF B cells and presented on B cell surface, antigen specific CD4^+^ T cells are activated. Then, RF B cells receive CD4^+^ T cell help via CD40 signaling or cytokines produced by CD4 T^+^ cells, thus differentiating into RF-producing plasma cells. To summarize, microbial antigens, B cell receptors, TLRs, and CD4^+^ T cells cooperate to activate RF-producing B cells after bacterial infection, and both B cell receptor signaling and CD4 T^+^ cell help are critical to enhance the RF secretion^32^. In our study, differential gene expressions found between *P. gingivalis*- and *T. denticola*-exposed mice, which had different levels of RF, were related to B cell receptor signaling pathways, B cell proliferation, activation, and differentiation, CD4^+^ T cell costimulation, and Th1 cytokine production. Our results alongside the aforementioned study support that different RF induction by different periodontal bacteria might depend on whether they can impact on B cell receptor signaling pathways, B cell proliferation, activation, and differentiation, and CD4^+^ T cell costimulation and cytokine production.

Rifkin and colleagues showed that the sera of autoimmune mice, but not that of nonautoimmune mice, induced activation of RF-producing B cells in vitro^33^. The stimulating factors were the IgG2a immune complexes that were present in the sera of autoimmune mice. However, not all immune complexes activate RF-producing B cells; a subsequent study showed that IgG2a immune complexes containing nucleosome antigens efficiently activated RF-producing B cells by engaging B cell receptors and TLRs, but IgG2a immune complexes containing other antigens failed^34^. These findings indicate that the activation of B cells expressing RF depends on the type of antigens present on the immune complexes.

This study has some limitations. First, only a relatively small number of patients were included in the subgingival microbiome sequencing analyses. Therefore, our results need to be validated in further studies within larger study population. However, we performed murine model experiments and single-cell RNA sequencing analyses to support our findings in microbiome sequencing analyses. Second, we did not include a healthy control group in this study. However, the distinct subgingival microbial compositions in patients with RA and at-risk individuals compared with healthy controls have been confirmed in previous studies^9–11^. Instead, we aimed to compare the subgingival microbial compositions among patients with RA according to their RF or RA status.

## Conclusion

We revealed the differential landscape of the subgingival microbiome among the different RF status in patients with RA and individuals at risk of RA. Oral infection with different periodontal bacteria species caused different levels of RF during arthritis development in an RA mouse model. We suggest that periodontal bacteria affect RF status by differential involvement in B cell receptor signaling, B cell proliferation, activation, and differentiation, and CD4^+^ T cell costimulation and cytokine production.

## Data availability 

The datasets generated and/or analysed during the current study are available in the National Center for Biotechnology Information (NCBI) BioProject repository, https://www.ncbi.nlm.nih.gov/bioproject/PRJNA765928 (metagenome sequencing datasets) and https://www.ncbi.nlm.nih.gov/bioproject/PRJNA820883 (single-cell RNA sequencing datasets).

## Supplementary Information

Supplementary Information 1.

Supplementary Information 2.
