# Supplementary material for: Differential effects of periodontal microbiome on the rheumatoid factor induction during rheumatoid arthritis pathogenesis
Source: Sci Rep. 2022 Nov 16;12:19636. doi: 10.1038/s41598-022-21788-y (PMC9668994; doi:10.1038/s41598-022-21788-y)
Supplement: Supplementary file 1 — Supplementary Information 1. [file 41598_2022_21788_MOESM1_ESM.pdf]

Online Supplementary Materials

Supplementary Table S1. Characteristics of patients with pre-RA, NORA, and chronic RA who underwent periodontal probing depth examination.

Supplementary Table S2. Characteristics of patients with pre-RA, NORA, and chronic RA who underwent subgingival microbiome analysis.

Supplementary Fig. S1. Comparison of periodontitis prevalence between patients with pre-RA (n = 18) and age-, sex-, body mass index-, smoking-, diabetes-, and hypertension-matched controls (n = 72).

Supplementary Table S1 Characteristics of patients with pre-RA, NORA, and chronic RA who underwent periodontal probing depth examination

Variable	Pre-RA (n=18)	NORA (n=18)	Chronic RA (n=49)	p value
Age, years	50.6 (11.5)	52.3 (12.9)	53.5 (10.6)	0.637
Female sex	88.9%	94.4%	89.8%	1.000
BMI, kg/m^2	22.3 (2.8)	21.5 (2.7)	22.6 (3.8)	0.565
Smoker	9.1%	28.6%	16.7%	0.733
PPD, mm	5.5 (4.8–7.3)	6 (5–6.3)	6 (5–7)	0.525
Periodontitis				
Mild (PPD >3 <5)	16.7%	11.1%	18.4%	0.531
Moderate (PPD ≥5 <7)	50.0%	61.1%	46.9%	
Severe (PPD ≥7)	27.8%	22.2%	34.7%	
Plaque index	47.1 (8.7)	42.6 (15.5)	41.9 (16.7)	0.590
Frequency of toothbrushing	2.5 (2–3)	3 (2–3)	2.5 (2–3)	0.774
Floss or intertooth brush	66.7%	73.3%	46.2%	0.131
RA duration, months	–	1 (1–3.3)	72 (48–150)	<0.001
ESR, mm/hr	21 (17.8–29)	38 (21–49.3)	19 (14–31)	0.016
CRP, mg/dL	0.04 (0.03–0.38)	0.22 (0.06–1.16)	0.12 (0.04–0.27)	0.217
RF, IU/mL	78.2 (41.3–161.7)	134 (29.8–218.6)	75.2 (24.9–353.5)	0.747
Positive RF	82.4%	94.1%	77.1%	0.390
ACPA, U/mL	30 (11.4–292.8)	279.5 (83.3–340)	103.5 (53.5–340)	0.083
Positive ACPA	88.9%	93.8%	100%	0.384
Medication				
Steroid	22.2%	44.4%	81.6%	<0.001
PD equivalent dose	5.0 (3.2–5.0)	5.0 (5.0–7.5)	5.0 (2.5–5.0)	0.176
Methotrexate	5.6%	38.9%	83.7%	<0.001
Biologic agents	0%	0%	20.4%	0.014

Pre-RA, preclinical rheumatoid arthritis; NORA, new-onset RA; BMI, body mass index; PPD, periodontal probing depth; ESR, erythrocyte sedimentation rate; CRP, C-reactive protein; RF, rheumatoid factor; ACPA, anti-citrullinated protein antibodies; PD, prednisone

Supplementary Table S2 Characteristics of patients with pre-RA, NORA, and chronic RA who underwent subgingival microbiome analysis

	RA status	Sex	Age	BMI	Smoking	RA duration	RF	ACPA	ESR, mm/hr	CRP, mg/dL	DAS28	PD equivalent	DMARDs
1	Pre-RA	F	66	25.2	No	–	High-positive	High-positive	30	0.21	3.77	–	–
2	Pre-RA	M	45	23.8	No	–	High-positive	High-positive	6	0.04	1.54	–	–
3	Pre-RA	F	33	16.2	No	–	High-positive	High-positive	38	0.42	3.11	5 mg	HCQ
4	Pre-RA	F	54	23.2	No	–	Negative	High-positive	7	0.02	1.65	–	–
5	Pre-RA	F	52	19.5	No	–	High-positive	High-positive	22	0.18	2.59	–	–
6	NORA	F	54	20.7	No	2 months	Low-positive	High-positive	8	0.06	1.74	7.5 mg	MTX
7	NORA	F	69	25.3	No	–	Negative	High-positive	81	6.73	5.6	–	–
8	Chronic RA	F	65	18.1	No	15 years	Negative	High-positive	20	0.02	2.38	2.5 mg	MTX+IFX
9	Chronic RA	F	68	21.0	No	18 years	Low-positive	High-positive	12	0.02	2.17	–	BA
10	Chronic RA	F	57	19.4	No	3 years	High-positive	High-positive	30	0.26	2.95	7.5 mg	MTX+TAC

Pre-RA, preclinical rheumatoid arthritis; NORA, new-onset RA; BMI, body mass index; RF, rheumatoid factor; ACPA, anti-citrullinated protein antibodies; ESR, erythrocyte sedimentation rate; CRP, C-reactive protein; DAS28, disease activity score of 28 joints; PD, prednisone, DMARDs, disease-modifying antirheumatic drugs; HCQ, hydroxychloroquine; MTX, methotrexate; IFX, infliximab; BA, bucillamine; TAC, tacrolimus

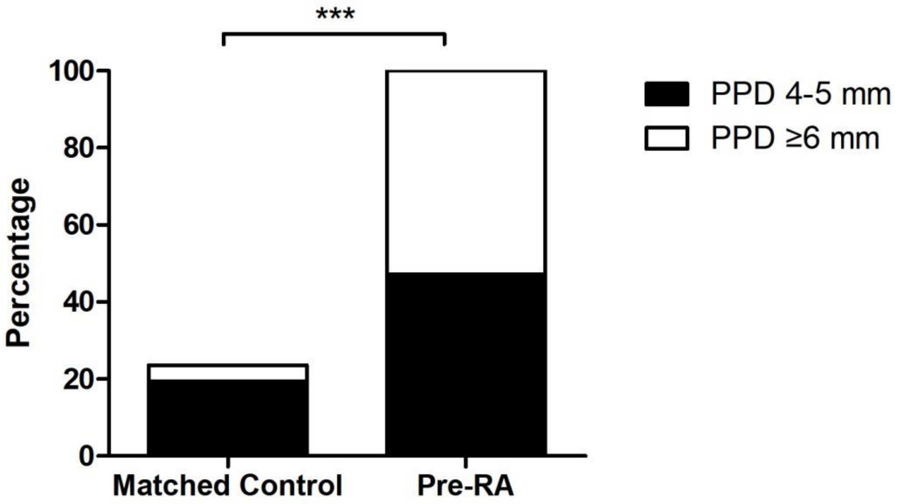

Supplementary Figure S1 Comparison of periodontitis prevalence between patients with pre-RA (n = 18) and age-, sex-, body mass index-, smoking-, diabetes-, and hypertension-matched controls (n = 72). Pre-RA, preclinical rheumatoid arthritis; PPD, periodontal probing depth.
